# Factors influencing self-management of adults living with HIV on antiretroviral therapy in Northwest Ethiopia: a cross-sectional study

**DOI:** 10.1186/s12879-020-05618-y

**Published:** 2020-11-23

**Authors:** Habtamu Areri, Amy Marshall, Gillian Harvey

**Affiliations:** 1 Adelaide Nursing School, Faculty of Health and Medical Sciences, The University of Adelaide, Adelaide, SA 5005 Australia; 2 School of Nursing and Midwifery, College of Health Sciences, Addis Ababa University, 4412 Addis Ababa, Ethiopia

**Keywords:** Adults living with HIV, Antiretroviral therapy, HIV/AIDS, Individual and family self-management theory, Knowledge of HIV treatment, Self-efficacy, Self-management

## Abstract

**Background:**

Effective self-management is an important consideration for adults living with HIV on therapy to enable people to maintain their health and well-being whilst living with chronic HIV. Although numerous attempts have been made to implement and improve HIV self-management practice, there is limited evidence on effective self-management strategies, particularly in sub-Saharan Africa. This study aimed to identify the level and factors influencing the self-management practice of adults living with HIV on antiretroviral therapy.

**Methods:**

A cross-sectional survey was conducted on a sample of 415 adults living with HIV on antiretroviral therapy at a major referral hospital in Northwest Ethiopia using convenience sampling. A theory of self-management – the Individual and Family Self-Management Theory - guided the study design, analysis and presentation of the data. A face-to-face survey tool was administered for data collection, and the data were entered and analyzed using SPSS version 25.0.

**Results:**

Over half (58.1%) of the respondents were female. Many of the respondents did not know their HIV stage (76.9%) but reported adequate knowledge of their treatment (79.5%). The mean self-management score was 1.94^+^ 0.22 out of a total score of 3. Female gender was associated with decreased self-management. Contextual factors (gender, educational level, job status, income, living in a rural area, and awareness of HIV stage) explained 8.2% of the variance in self-management. The explanatory power increased by 9.2% when self-management process variables (self-efficacy, setting a goal, knowledge of antiretroviral therapy, HIV disclosure, and use of reminders) were added. Intervention-focused variables (encouraging disclosure and adherence support) increased the proportion of explained variance by 2.3%.

**Conclusions:**

The findings of the study indicate that the level of self-management practice amongst the population studied was low compared to international literature. Our study findings support the theoretical model and previously identified factors influencing HIV self-management. The most important predictors of lower self-management practice in Ethiopia were female gender, illiteracy, lack of awareness of HIV stage, low self-efficacy, absence of reminders, lack of encouragement to disclose and absence of adherence support. HIV care providers should seek ways to empower and support adults living with HIV to self-manage, particularly through enhancing self-efficacy and encouraging the use of reminders.

**Supplementary Information:**

The online version contains supplementary material available at 10.1186/s12879-020-05618-y.

## Background

Globally, since the start of the HIV epidemic, it is estimated that over 70 million people have been infected and 32 million have died of AIDS-related illnesses. About 38 million people are currently living with HIV, most of whom are adults (36.2 million) [1]. This figure includes around 25.7 million individuals in Africa [2], of whom 690,000 are living in Ethiopia. Women are disproportionately represented in Ethiopia, with approximately 410,000 out of the 690,000 being women. Even though rates of new HIV infection declined by 79% from 2010 to 2018, 11,000 people died from AIDS-related complications in Ethiopia in the year 2018. The national Antiretroviral Therapy (ART) coverage is 66%, meaning around a third of the population currently lack access to ART [3].

Recently, there has been an emphasis on self-management outcomes to achieve optimal benefits from HIV interventions in Ethiopia [4]. Self-management refers to managing illness need (managing medication, daily physical health), activating social support (family, peers, and HIV care providers), and living with chronic illness [5, 6]. Successful ART programs depend on effective and prolonged SM programs to ensure optimal uptake of medical, physical, emotional, and psychological recommendations [5]. Health care providers can help identify the unique SM needs of patients and guide a patient-centred approach to enable patients to be the self-manager of their health conditions [6–8].

Studies on HIV SM in high-income and upper-middle-income countries focus on three domains of SM, namely daily physical health practice, social support, and living with HIV [6, 9, 10]. Daily physical health practice refers to physical exercise, diet management, reducing stress, symptom management, managing drug side effects and implementing other recommended physical health activities. Activating social support refers to the use of families, health care providers and social networking for effective SM. Living with HIV refers to accepting and adjusting to HIV, dealing with stigma and giving meaning to life with existing conditions [5, 6, 10]. A survey conducted among women living with HIV in the United States of America (USA) reported mean scores of daily physical health practice 2.19 ± 0.53, activating social support 2.0 ± 0.88, living with chronic HIV 2.64 ± 0.43 and an overall mean SM score of 2.28 ± 0.61 out of a maximum score of 3, which was interpreted by the authors as a moderate level of SM [11]. A similar study using the same scale and conducted in China indicated lower mean scores: daily physical health practice, 1.80 ± 0.42, activating social support, 1.47 ± 0.63, living with chronic HIV, 2.46 ± 0.43 and an overall mean SM score of 1.91 ± 0.36, which was interpreted as low [12]. A third study conducted in Korea using the same scale reported an overall SM mean score of 2.00 ± 0.49, again interpreted as low [9]. These findings demonstrate variable levels of SM across different international settings.

**Fig. 1:**
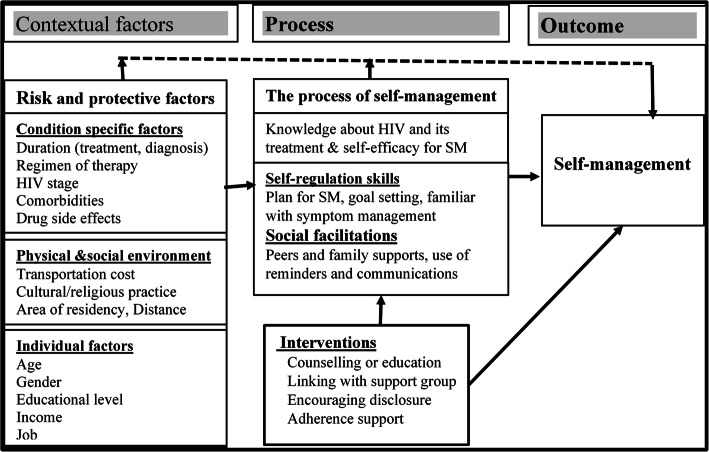
Conceptual framework of self-management of ALWHIV developed from IFSMT

The factors influencing SM will be discussed through the theoretical lens of the Individual and Family Self-management Theory (IFSMT) which was developed to frame the management of chronic conditions including HIV [13]. This middle-range theory is descriptive and comprises dynamic and interrelated constructs defined as the context, the process of SM, SM interventions and SM outcomes. The contextual factors focus on “risk and protective factors”, including condition-specific factors, the physical and social environment, and individual factors. The “process of SM” encompasses knowledge of ART, self-efficacy, self-regulation abilities, and social facilitation. Self-regulation is a process of individuals setting an engagement to achieve a change in health behaviours. Social facilitation is a means of social influence, support, and negotiated collaboration. Social influence can be exerted by HIV care providers, family, and peer networks with the explicit goal of facilitating engagement in recommended behaviours. SM interventions are interventions intended to enhance both the process and outcomes of SM [13]. SM outcomes are concerned with different aspects of practicing SM and include daily physical practice, activating social support, and managing medication whilst living with chronic HIV [5, 6, 9, 11, 12, 14] (see Fig. 1).

Few studies have addressed all the domains of SM in a comprehensive way, as outlined in the IFSMT [9, 11, 12]. Rather, individual studies tend to focus on specific aspects of SM and the factors that affect this, such as treatment duration [15], regimen complexity [16, 17], comorbidities [16, 18–20], and drug side effects [21], which can be categorized as condition-specific factors under the contextual factors in IFSMT. Other contextual factors studied that influence SM of ALWHIV correspond with the physical and social environment domains of the IFSMT. These include the cost of travel [22], area of residence and distance from health facilities [12], and religious practices such as fasting or using holy water [23]. In the IFSMT, individual characteristics are also viewed as responsible for enhancing or inhibiting engagement in SM behaviours. Individual factors influencing SM identified in previous research include age, gender, educational level [11] and income [11, 12, 24].

According to the IFSMT, the process of SM can be influenced by factors such as knowledge, self-efficacy, self-regulation abilities, and social facilitation. Russell et al. [5, 25] and WHO [26] reported that adequate knowledge of HIV treatment enhances SM. Self-efficacy, namely a person’s perception of their ability to self-manage, is another factor that influences SM [27], with studies demonstrating that a higher self-efficacy is associated with improved utilization of recommended health behaviours [28, 29]. The presence of social support [12, 30], reminders and a good relationship with HIV care providers have been shown to facilitate engagement in care [31, 32]. However, barriers to mobilizing existing community resources for SM include non-disclosure of HIV status, inadequate information, and negative community perceptions towards people living with HIV. Therefore, interventions are required to overcome the barriers and enhance SM practice [30, 32]. Examples of such interventions include counselling, adherence support and encouraging disclosure [30, 31]. Overall, patient-centred interventions are seen to be the most effective in fostering engagement in SM practice [13, 31–33].

In Ethiopia, it has proved difficult to avoid HIV related complications, which are a cause of frequent hospital visits [34, 35]. Sub-optimal levels of adherence, fear of stigma and disclosure, and opportunistic infection treatment costs remain a major challenge for ALWHIV in Ethiopia [36–38]. Although the chronic care model has been incorporated and implemented in the HIV program of the country, there is a lack of evidence about the practice of SM for ALWHIV on ART. Therefore, this study aimed to identify the level and factors influencing the SM of ALWHIV on ART in a regional area of Ethiopia. The study aimed to address two specific objectives, namely: 1) to determine the level of SM practice and 2) to identify factors influencing SM practice of ALWHIV on ART in the study area.

## Methods

### Study design, setting, participants and sampling

In order to address the research question, a survey was conducted at a referral hospital in Northwest Ethiopia. The study sample was selected from ALWHIV (18 years plus), enrolled in ART care for at least six months and visiting the referral hospital during the study period (April 1 to May 30, 2019). The study area had 3802 adults on ART (2243 females and 1559 males) in the year 2018 [39]. Daniel’s [40] “single population proportion formula” was used to estimate the sample size. The estimation was based on an assumption of the standard score of 95% (1.96), the sampling error 0.05, and a 50% of SM practice among ALWHIV since no previous study had been conducted in the study area. This indicated a required sample size of 384. To allow for a 10% non-response rate, the final sample size was set at 422. Convenience sampling was used to identify 12–15 volunteer participants per day attending the HIV clinic.

#### Variables and measurement

The survey tool comprised a combination of existing instruments and additional survey questions that were designed for the study based on the constructs of IFSMT and relevant literature (Table 1 and Additional file 2).

**Table 1:** Summary of the survey instruments

Theoretical constructs	Details of research instrument/s used
Contextual factors	21 item questionnaire developed for this research:
Sociodemographic: individual factors	5 items
Sociodemographic: physical and social environment	7 items
Clinical: Conditions specific factors	9 items
Process of SM	
Knowledge of ART	Existing 10 item questionnaire [41]
Self-efficacy for SM	Existing 8 item questionnaire [27]
Self-regulation skills	3 item questionnaire developed for this research
Social facilitation	5 item questionnaire developed for this research
SM interventions	3 Item questionnaire developed for this research
Outcome	
SM practice (20 items)	Existing 20 item questionnaire [10]

### Contextual factors

Sociodemographic and clinical data from the participants were collected using a 21-item questionnaire developed for the purposes of the study, based on the IFSMT and variables identified in the literature. Sociodemographic questions encompassed the individual, physical and social environment. Clinical factors influencing the patient’s health status included duration of HIV diagnosis, duration of ART, HIV stage, treatment changes, comorbidities, other treatments, number of drugs taken, and drug side effects.

### The process of SM

The process of SM was assessed across 4 areas, namely knowledge of ART, self-efficacy, self-regulation abilities, and social facilitation, using a combination of existing instruments and additional questionnaires developed specifically for the study.

The *knowledge of ART* was measured using an existing instrument*.* The tool consisted of ten items answered as ‘yes’ or ‘no’ responses. Based on the responses, knowledge level was categorized as adequate (6–10) or inadequate (0–5) [41, 42]. The *self-efficacy* was measured using an existing perceived medical condition SM scale. Overall scores were calculated by obtaining the mean of the 8 items scored over a 3-point Likert scale *(1 = disagree, 2 = neutral, and 3 = agree)*. Final scores ranged from 8 to 24, with higher scores reflecting greater self-efficacy [27]. Based on the IFSMT and existing literature, three survey items were developed to assess *self-regulation skills* such as goal setting, planning, and symptom management. These items were answered as “yes” or “no” responses. Similarly, based on the IFSMT and existing literature, five survey items were developed to assess *social facilitation*, which consisted of social influence and support from HIV care providers, family, peers and social networking. The items were answered as ‘yes’ or ‘no’ responses.

### SM interventions

SM interventions are strategies used by HIV care providers to enhance the SM practice of ALWHIV. Supportive interventions include information on drug management, symptom management, self-regulation enhancement and social networking. The study used different items to measure SM interventions related to counselling, encouragement to disclose HIV status and adherence support (answered by a yes/no response).

### Self-management

SM practice was measured using the HIV SM scale, originally developed in the USA to measure SM of women living with HIV [10]. The scale consists of 20 items that focus on daily physical health practice, activating social support and living with chronic HIV conditions. Each item is scored on a four-point Likert scale ranging from 0 to 3 (0 = *not applicable*, 1 = *none of the time*, 2 = *some of the time,* and 3 = *all the time)*. A higher score indicating better SM. Cronbach’s alpha ranges from 0.62–0.77 in the current study, which is slightly lower than the original study’s alpha value of 0.72–0.86 [10].

The current study translated the above existing tool [10] into the local language (Amharic) to combine them with the other sections created specifically for this study in Amharic. The locally translated data collection tool was pretested on 5% of the total sample size one month before the main data collection commenced, and some minor revisions were made. The data collection was conducted by degree qualified nurses with experience in research and HIV care. The survey was administered via a face-to-face interview in a private room with consented participants.

#### Statistical methods

The SM score out of a maximum of 60 was calculated and changed to mean score within SPSS version 25.0. There were no identified missing data. Continuously measured variables were summarized using the mean and standard deviation, while categorical items were described using frequencies and percentages. The difference in mean of SM was assessed using an Independent *t-test* and *one-way* ANO VA. Associations between selected factors and the outcome were assessed using Pearson and Spearman rank correlations, and Hierarchical multiple regression for binary and continuous outcomes, respectively. Variables that were statistically significant in bivariate analysis were used as covariates. Hierarchical multiple regression analysis was then used to identify factors influencing SM based on the IFSMT. Variables from contextual factors were entered at model 1.Variables from the process of SM were then added to the model at model 2, and finally, variables from SM interventions were added at model 3. A *p*-value of less than 0.05 was considered statistically significant.

## Results

The response rate to the survey was 98.34% (*n* = 415). The results are presented according to the key constructs of the IFSMT, namely: contextual factors (sociodemographic and clinical characteristics), the process of SM, SM interventions and SM outcomes. Findings from the inferential analysis are then presented to describe the factors influencing SM.

### Contextual factors

#### Sociodemographic: individual factors, physical and social environment

Over half, 58.1% (*n* = 241), of respondents were female, which is representative of the proportion of women living with HIV in Ethiopia. The mean age of the respondents was 41.05 ± 10.54 years. Over one-third of the respondents, 34.9% (*n* = 145) were illiterate, and over half, 51.3% (*n* = 213) were employed privately. The mean monthly income of the respondents was 100 ± 96.52USD. The mean distance travelled to the ART facility was 31.75 ± 41.11 km, and the cost of transportation was reported as a challenge for 29.6% (*n* = 123) of respondents. There was a difference in the mean of SM in terms of gender (males better in SM), educational level (higher educational level increased SM), job categories (those who were employed in government jobs were better in SM), and place of dwelling (urban dwellers had better SM than rural) (see Table 2).

**Table 2:** Mean SM according to demographic and clinical characteristics (*n* = 415)

Variables	***n*** (%)	Mean (SD)	t or F	***p***
**Gender**				
Male	174 (41.9)	39.54 (4.49)	2.829	0.005
Female	241 (58.1)	38.32 (4.32)		
**Educational level**				
Illiterate	145 (34.9)	37.72 (4.15)	6.279	< 0.001
Primary education	103 (24.8)	38.83 (4.40)		
High school ^a^	100 (24.1)	39.65 (4.21)	(a < b)	
College and above education ^b^	67 (16.1)	40.06 (4.58)		
**Job-status**				
Governmental employed ^a^	73 (17.6)	40.47 (4.17)	7.446	0.001
Privately employed ^b^	213 (51.3)	38.76 (4.28)		
No regular job	129 (31.1)	38.04 (4.44)	(a > b)	
**Religion**				
Orthodox Christian	396 (95.4)	38.87 (4.39)	0.621	0.538
Protestant Christian	6 (1.4)	39.33 (5.13)		
Muslim	13 (3.1)	37.54 (3.67)		
**Marital status**				
Never married	26 (6.3)	38.73 (4.15)	1.765	0.173
Married	208 (50.1)	39.23 (4.29)		
Live separated/Divorced/Widowed	181 (43.6)	38.40 (4.50)		
**Living arrangement**				
Lives alone	91 (21.9)	39.13 (4.56)	0.728	0.467
Lives with families/parents	324 (78.1)	38.75 (4.33)		
**Area of residency**				
Rural	106 (25.5)	38.00 (3.94)	−2.289	0.023
Urban	308 (74.5)	39.12 (4.49)		
**HIV Stage**				
I know it	98 (23.6)	40.47 (4.47)	4.312	< 0.001
I do not know it	317 (76.4)	38.33 (4.23)		
**Treatment changed**				
Yes	110 (26.5)	39.13 (4.20)	0.813	0.417
No	305 (73.5)	38.73 (4.45)		
**Comorbidities**				
Yes	45 (10.8)	38.76 (4.54)	−0.131	0.896
No	370 (89.2)	38.85 (4.37)		
**Treatment for comorbidities**				
Yes	40 (9.6)	38.83 (4.59)	−0.017	0.987
No	375 (90.4)	38.84 (4.36)		
**Number of drugs**				
One type	312 (75.2)	38.84 (4.37)	0.345	0.709
Two types	68 (16.4)	39.09 (4.88)		
More than two types	35 (8.4)	38.32 (3.34)		
**Drug side effects**				
Yes	38 (9.2)	38.53 (4.27)	−0.457	0.648
No	377 (90.8)	38.87 (4.40)		

^a & b^ post hoc analysis significant mean difference

#### Clinical characteristics: condition-specific factors

The mean duration of HIV diagnosis and therapy was 8.69 ± 3.89 and 7.99 ± 3.66 years, respectively. Over three-quarters, 76.4% (*n* = 317) of the respondents did not know their HIV stage. Respondents who knew their HIV stage had a higher mean SM score (see Table 2).

### The process of SM

#### Knowledge, self-efficacy, self-regulation skills, and social facilitation

The majority, 79.5% (*n* = 330), of the respondents reported adequate knowledge of their HIV treatment. The mean self-efficacy score was 19.76 ± 0.12 (out of a possible total of 24). Most of the respondents, 93.0% (*n* = 386), reported that they got information on ART from health care providers, and over half, 51.6% (*n* = 214), received reminders about SM. A higher mean SM score was observed among respondents who had adequate knowledge, set a plan for emotional distress*,* were familiar with how to manage HIV-related symptoms, had set goals in relation to their treatment program*,* who received information on ART and who used reminders for SM. The majority, 74.2% (*n* = 308), of respondents did not have support either from family or peers (see Table 3). One fifth, 20.7% (*n* = 86) of the participants did not disclose their HIV status to the community for reasons relating to fear of stigma (47.3%; *n* = 79), fear of discrimination (46.7%; *n* = 78), or because they did not consider it to be important (6.0%; *n* = 10).

**Table 3:** Mean SM according to process of SM and SM interventions (*n* = 415)

Variables	***n*** (%)	Mean (SD)	t or F	***p***
**The process of SM**				
**Knowledge of ART**				
Inadequate	85 (20.5)	37.73 (3.81)	2.631	0.009
Adequate	330 (79.5)	39.12 (4.48)		
**Self-efficacy for SM, Mean (SD)**	19.76 ± 0.12			
**Have a plan for emotional distress**				
Yes	358 (86.3)	39.04 (4.38)	2.379	0.018
No	57 (13.7)	37.56 (4.18)		
**Familiar with how to manage HIV illness-related symptoms**				
Yes	165 (39.8)	39.38 (4.59)	2.070	0.039
No	250 (60.2)	38.48 (4.21)		
**Set a goal in the process of HIV therapy**				
Yes	395 (95.2)	38.94 (4.35)	2.193	0.029
No	20 (4.8)	36.75 (4.59)		
**Got support from family/peers**				
Yes	107 (25.8)	39.48 (4.81)	2.380	0.018
No	308 (74.2)	38.56 (4.22)		
**Got information on ART from health care providers**				
Yes	386 (93.0)	38.96 (4.39)	2.041	0.042
No	29 (7.0)	37.24 (4.02)		
**Used reminders for HIV management**				
Yes	214 (51.6)	39.51 (4.37)	3.268	< 0.001
No	201 (48.4)	38.12 (4.29)		
**HIV disclosure**				
Yes	329 (79.3)	39.05 (4.31)	1.967	0.050
No	86 (20.7)	38.01 (4.55)		
**SM interventions**				
**Counselling was adequate for the next steps of treatment**				
Yes	335 (80.7)	38.91 (4.45)	0.678	0.498
No	80 (19.3)	38.54 (4.08)		
**Adequately linked to social networks**				
Yes	112 (27.0)	39.48 (4.40)	1.949	0.052
No	303 (73.0)	38.56 (4.35)		
**Got support from adherence supporters**				
Yes	94 (22.7)	39.78 (4.81)	2.380	0.018
No	321 (77.3)	38.56 (4.22)		
**Encouraged to disclose HIV status**				
Yes	383 (92.3)	39.00 (4.34)	2.785	0.006
No	32 (7.7)	36.78 (4.39)		

### Self-management interventions

The majority, 80.7% (*n* = 335), of respondents considered the counselling they received was adequate for their next step of HIV treatment. Most of the respondents, 92.3% (*n* = 383), were encouraged to disclose their HIV status. Almost three-quarters of the respondents, 73.0% (*n* = 303), were not linked to social networks. A higher mean SM score was reported among respondents who were encouraged to disclose their HIV status and those who received adherence support (see Table 3).

### Outcome: self-management practice

The overall SM score (out of 60) was converted to a score out of 3, in line with the Likert scale used and in order to make comparison with reported from other studies using the same instrument. The overall mean of the HIV SM item score was 1.94 ^+^ 0.22 out of maximum 3. About 47% (*n* = 195) of the participants scored below the mean value whereas the rest, 53%(*n* = 220) scored above the mean value of total SM practice. The mean level of SM outcomes varied across the domains as follows: daily physical health practice mean score was 1.95 ^+^ 0.23; activating social support mean score was 1.64^+^ 0.45; living with chronic HIV mean score was 2.11^+^ 0.56.

### Correlational analysis

Variables positively related to SM scores were educational level, job status, income, knowledge of ART, self-efficacy, setting goals, use of reminders, encouragement to disclose HIV status, receipt of adherence support, and disclosure of HIV status. Gender and living area were negatively associated with SM (see supplementary file 1).

### Factors influencing self-management practice

Hierarchical multiple regression analysis was used to identify factors influencing SM based on the theoretical model after testing for fitness. In step 1, statistically significant contextual variables identified in the bivariate analysis were entered (see supplementary file 1). Step 1 revealed that the statistically significant contextual factors influencing SM explained 8.2% of the variance in SM. Self-awareness of HIV stage was a contributor to the prediction (*β = 0.14, p = 0.008*). In step 2, statistically significant SM process variables were added, and explanatory power increased by 9.2%. Self-efficacy and the use of reminders were the most significant variables in predicting SM. In step 3, statistically significant intervention-focused variables were added, and explanatory power increased by 2.2%. The final model explained 19.7% of the variance in SM of ALWHIV on ART and was statistically significant, adjusted *R*^2^ = 17.1% *F (13, 401) = 7.552, p < 0.0005*). Self-efficacy (*β = 0.20, p < 0.0005*) and the use of reminders (*β = 0.15, p = 0.002*) were the strongest predictors of SM (Table 4).

**Table 4:** Hierarchical multiple regression of factors influencing SM of ALWHIV on ART

	Self-management								
***Variables***	***Model 1***			***Model 2***			***Model 3***		
	**B**	**β**	***t(p)***	**B**	**β**	***t(p)***	***B***	***β***	***t(p)***
(Constant)	38.26		71.45 (0.000)	27.95		14.52 (0.000)	25.93		13.10 (0.000)
Gender (female)	−0.79	−0.09	−1.81 (0.071)	− 0.74	− 0.08	− 1.75 (0.081)	− 0.90	− 0.10	− 2.13 (0.034)
Educational level (formal)	0.83	0.09	1.76 (0.089)	0.90	0.10	1.89 (0.059)	0.95	0.10	2.02 (0.044)
Income (mean)	6.11^e-5^	0.04	0.65 (0.52)	−8.82^e-6^	−0.01	−0.10(.923)	3.97^e-5^	0.02	0.44 (0.662)
Job status (employed)	0.84	0.07	1.30 (0.196)	0.85	0.07	1.37 (0.172)	1.01	0.09	1.64 (0.101)
Living area (rural)	−0.65	−0.06	−1.31 (0.190)	−0.33	− 0.03	−0.70 (0.487)	0.00	0.00	−0.01 (0.999)
Knowledge of HIV stage	1.40	0.14	2.66 (0.008)	1.14	0.11	2.24 (0.026)	1.00	0.10	1.99 (0.047)
Knowledge of ART				0.42	0.04	0.82 (0.414)	0.49	0.05	0.97 (0.334)
Self-efficacy				0.38	0.21	4.55 (0.000)	0.37	0.20	4.43 (< 0.001)
Disclosed HIV status				1.14	0.11	2.26 (0.024)	0.98	0.09	1.97 (0.049)
Set goal for SM				1.52	0.07	1.63 (0.103)	1.51	0.07	1.62 (0.105)
Use of reminder				1.41	0.16	3.46 (0.001)	1.30	0.15	3.20 (0.002)
Encouraged to disclose HIV status							1.64	0.10	2.15 (0.032)
Adherence support							1.13	0.11	2.31 (0.022)
R^2^ (Adjusted R^2^)	0.082 (0.069)			0.175 (0.152)			0.197 (0.171)		
F *(df)*	6.110 (6408)			7.764 (11,404)			7.552 (13,401)		
p	< 0.0005			< 0.0005			< 0.0005		
Δ R^2^	0.082			0.092			0.022		
F (*df*)	6.110 (6408)			9.027 (5403)			5.446 (2400)		
*P*	< 0.0005			< 0.0005			0.005		

Durbin-Watson statistic = 1.799.

## Discussion

Several international studies on SM of ALWHIV have been conducted using the same measure of SM practice that was applied in this research [9, 11, 12]. However, evidence to date has been lacking on SM and influencing factors amongst ALWHIV in low-income countries such as Ethiopia. Table 5 summarises the level of SM at an overall level and by each of the three-domain scores in the Ethiopian sample compared to results from studies in the United States of America (USA, female-only sample), Korea and China.

**Table 5:** Level of self-management in this study and different literature

	Ethiopia	USA (females only) [11]	Korea [9]	Chin a[12]
Sample size	415	260	203	322
Overall SM score	1.94	2.28	2.00	1.91
Daily physical health practice	1.95	2.19	1.92	1.80
Mobilising social support	1.64	2.00	1.66	1.47
Living with chronic HIV	2.11	2.64	2.42	2.46

Similar patterns of scores are apparent across all four countries, with activating social support being the lowest of the domain scores and living with chronic HIV the highest score. However, overall SM scores are notably lower in the Ethiopian and Chinese studies, when compared to the USA [11] and Korea [9]. In these latter two study areas, the implementation of the SM model was earlier compared to the current study area, where it is relatively new. (Table 5).

The components of social support are multiple and require the input of different groups, including families, health care providers and peer supporters [13, 43]. Social support also depends on the availability of social services and the acceptance of the individual to receive support [10]. The findings of this study also show that the use of reminders can be an enabling factor for SM. These findings are consistent with surveys conducted in the USA [11] and China [12]. Both a systematic review [32] and the IFSMT [13] have identified the importance of social facilitation in SM. The level of daily physical health practice in this study is consistent with the Korean [9] study but lower than the study conducted in the USA [11] and higher than the study conducted in China [12]. In terms of daily physical health practice, a review on interventions to improve SM showed improvement amongst individuals who received skills training and education on the importance of daily physical health practice [32]. The mean score of living with chronic HIV in the study area is lower than studies conducted in the USA [11], Korea [9] and China [12] (Table 5), which may reflect the later implementation of the chronic care model in Ethiopia.

The study identified different factors influencing overall SM of ALWHIV on ART. Some of the variables are modifiable, whereas others are not, for example, gender and age. In this study, there was no age-related difference in SM, similar to the studies conducted in Korea [9] and China [12]. The study also identified potentially modifiable factors influencing SM. These included the educational level of ALWHIV, as higher educational levels showed an association with increased SM, a finding that is consistent with surveys conducted in the USA [11] and China [12]. This study showed a mean difference in SM between participants from rural and urban areas, with urban-dwelling participants having a higher level of SM. This could be because stigma and discrimination towards people living with HIV in rural communities remains a problem [36]. As a result of these fears, ALWHIV may not be willing to expose themselves to the community and not ready to mobilize the available supports.

SM is higher amongst ALWHIV who are aware of their HIV stage. This is consistent with previous literature that suggests a basic awareness of the HIV condition and its treatment helps in improving SM [5, 26]. A higher score of self-efficacy was associated with an increased level of SM, again in line with previous studies [29, 44, 45]. Overall, enhanced self-efficacy is an enabling factor in terms of retention in an HIV care program and success of HIV management [31]. However, evidence is lacking on the specific factors that determine self-efficacy for SM. This is an important area for future investigation as it is a key factor in the process of enhancing SM.

In this study, respondents who received adherence support, used reminders and were encouraged to disclose their HIV status reported higher levels of SM. These findings support the propositions of the Individual and Family Self-management Theory [13]. Previous research also supports the use of reminders and social support as important enabling factors for SM [12, 13, 31]. ALWHIV are in different social situations, and they need situation-specific support for successful engagement in care and treatment. In summary, SM requires strong social facilitation and SM interventions. Interventions to improve SM in Ethiopia should seek to address the most significant modifiable factors, for example, through framing practice with the IFSMT theoretical model and using the constructs of the theory as an ‘aide-memoire’ or checklist during follow up visits of patients.

### Limitations of the study

Despite the contribution, there were some limitations to the study. The respondents were from one referral hospital only, and the sampling technique used was convenience sampling. This could potentially limit the generalizability of the study findings to other settings, although the large sample size and high response rate enhance the level of confidence in the findings. Finally, the study findings could be subject to social desirability bias, as data collection involved the use of an interviewer-administered, self-report survey.

## Conclusions

The study found a relatively low level of SM amongst ALWHIV in Ethiopia. Factors influencing the level of SM were consistent with previous literature. The most important predictors of low SM in Ethiopia were female gender, illiteracy, lack of awareness of HIV stage, low self-efficacy, absence of reminders, lack of encouragement to disclose and absence of adherence support. Self-efficacy and the use of reminders were the most significant contributors to the prediction of SM scores. HIV care providers should evaluate the SM behaviours of ALWHIV and develop person-centred care plans and interventions that take account of important contextual and process-related influencing factors. This would facilitate the development of more tailored approaches, for example, to enhance self-efficacy and the appropriate use of reminders. Future qualitative research is recommended to develop a more in-depth understanding of the barriers and enablers of effective SM of ALWHIV in the study area and help inform the design of future tailored interventions.

### Implications for practice

The study is the first of its kind in Africa, where HIV prevalence is high, and health care access and infrastructure are limited. Therefore, this study could be a baseline for policymakers, implementers and researchers in the region. It highlights to policymakers the importance of addressing SM in chronic HIV. Many of the factors are modifiable, although addressing them will require input from both HIV care providers and patients. HIV care providers should work on enhancing self-efficacy, as it is a crucial factor in HIV care and treatment. HIV care providers should also motivate and help patients to build self-belief and confidence to practice SM. Increased self-efficacy could help ALWHIV to mobilize existing support, manage symptoms, and cope with the illness. It is also important to devise a mechanism for enhancing the use of reminders, for example, through use of technology-assisted solutions such as phone calls, text messaging and online support, and other social facilitation approaches for improving the SM of ALWHIV on ART. HIV care providers should identify the target population based on contextual and process factors in the design and delivery of SM programs. This could be enhanced by considering the constructs of the IFSMT when working with ALWHIV on ART.

## Supplementary Information

**Additional file 1 **: **S1 Table 5**. correlational analysis between different predictors and self-management outcomes

**Additional file 2 **: **S2**. Survey tool

## Data Availability

The datasets used and/or zanalyzed during the current study are available from the corresponding author on reasonable request.

## Abbreviations

AIDS: Acquired Immunodeficiency Virus
ART: Antiretroviral therapy
ALWHIV: Adults Living with HIV
HIV: Human Immunodeficiency Virus
IFSMT: Individual and Family Self-Management Theory
SM: Self-Management
WHO: World Health Organization
