# Supplementary material for: Factors influencing self-management of adults living with HIV on antiretroviral therapy in Northwest Ethiopia: a cross-sectional study
Source: BMC Infect Dis. 2020 Nov 23;20:879. doi: 10.1186/s12879-020-05618-y (PMC7686766; doi:10.1186/s12879-020-05618-y)
Supplement: Supplementary file 1 — Additional file 1 : S1 Table 5. correlational analysis between different predictors and self-management outcomes [file 12879_2020_5618_MOESM1_ESM.docx]

**S1: Correlational analysis between different predictors and self-management outcomes**

| **Contextual factors** | | **1** | **2** | **3** | **4** | **5** | **6** | **7** | **8** | **9** | **10** | **11** | **12** | **13** | **14** | **15** | **16** | **17** | **18** | **19** | **20** | **21** | **22** | **23** |
| --- | --- | --- | --- | --- | --- | --- | --- | --- | --- | --- | --- | --- | --- | --- | --- | --- | --- | --- | --- | --- | --- | --- | --- | --- |
| **1** | Gender | 1 |  |  |  |  |  |  |  |  |  |  |  |  |  |  |  |  |  |  |  |  |  |  |
| **2** | Age | **-.34^**^** | 1 |  |  |  |  |  |  |  |  |  |  |  |  |  |  |  |  |  |  |  |  |  |
| **3** | Education | **-.11^*^** | .09 | 1 |  |  |  |  |  |  |  |  |  |  |  |  |  |  |  |  |  |  |  |  |
| **4** | Job status | **-.21^**^** | .06 | **.32^**^** | 1 |  |  |  |  |  |  |  |  |  |  |  |  |  |  |  |  |  |  |  |
| **5** | Income | **-.23^**^** | .08 | **.25^**^** | .**43^**^** | 1 |  |  |  |  |  |  |  |  |  |  |  |  |  |  |  |  |  |  |
| **6** | Area of living | .01 | -.03 | **-.23^**^** | -.07 | .03 | 1 |  |  |  |  |  |  |  |  |  |  |  |  |  |  |  |  |  |
| **7** | Distance | -.09 | .07 | -0.03 | .09 | **.19^**^** | **.42^**^** | 1 |  |  |  |  |  |  |  |  |  |  |  |  |  |  |  |  |
| **8** | Cost | .01 | .00 | **-.18^**^** | **-.16^**^** | **-.23^**^** | **.24^**^** | .**37^**^** | 1 |  |  |  |  |  |  |  |  |  |  |  |  |  |  |  |
| **9** | HIV stage | **-.12^*^** | .08 | **.30^**^** | **.22^**^** | **.20^**^** | **-.16^**^** | -.01 | **-.11^*^** | 1 |  |  |  |  |  |  |  |  |  |  |  |  |  |  |
| **10** | Other treatment | .03 | .07 | .08 | -.07 | -.05 | -.04 | .02 | **.13^**^** | .03 | 1 |  |  |  |  |  |  |  |  |  |  |  |  |  |
| **11** | No. of drugs | **-.12^*^** | .08 | **-.12^*^** | -.09 | -.07 | **.14^**^** | **.17^**^** | **.24^**^** | -.02 | **.14^**^** | 1 |  |  |  |  |  |  |  |  |  |  |  |  |
| **12** | Side effects | -.09 | -.02 | .03 | .10 | -.04 | -.05 | -.05 | -.01 | -.02 | **.12^*^** | **.15^**^** | 1 |  |  |  |  |  |  |  |  |  |  |  |
|  | **Process of SM** |  |  |  |  |  |  |  |  |  |  |  |  |  |  |  |  |  |  |  |  |  |  |  |
| **13** | Knowledge | **-.13^**^** | .06 | **.16^**^** | **.14^**^** | **.19^**^** | -.09 | -.04 | **-.12^*^** | **.13^**^** | .07 | .00 | .00 | 1 |  |  |  |  |  |  |  |  |  |  |
| **14** | Self-efficacy | **-.11^*^** | **.13^**^** | **.10^*^** | **.10^*^** | **.16^**^** | **-.12^*^** | .03 | -.05 | .06 | -.02 | -.07 | -.**15^**^** | .04 | 1 |  |  |  |  |  |  |  |  |  |
| **15** | Plan for emotion | **-.10^*^** | **.04** | **.12^*^** | **.11^*^** | .08 | -.01 | -.03 | -.02 | .09 | -.01 | .05 | .03 | .09 | .**10^*^** | 1 | . |  |  |  |  |  |  |  |
| **16** | Familiar with symptom mgt. | **-.16^**^** | **.14^**^** | **.25^**^** | **.17^**^** | **.13^*^** | -.07 | -.05 | **-.11^*^** | **.14^**^** | -.03 | -.07 | **.10^*^** | **.11^*^** | .00 | **.13^*^** | 1 |  |  |  |  |  |  |  |
| **17** | Set a goal | .01 | -.01 | .01 | .02 | .00 | -.05 | .01 | -.05 | .02 | .00 | .03 | -.01 | .08 | .07 | .**24^**^** | -.02 | 1 |  |  |  |  |  |  |
| **18** | Uses of reminders | .08 | -.07 | -.07 | -.03 | .07 | -0.03 | -.09 | -.09 | **.11^*^** | **-.11^*^** | .06 | -.08 | .09 | -.03 | -.07 | -.04 | .03 | 1 |  |  |  |  |  |
| **19** | Disclosed HIV | .**16^**^** | .01 | -.03 | -.08 | -.07 | **-.11^*^** | **-.17^**^** | -.01 | -.05 | .07 | .05 | .06 | .05 | .05 | .06 | -.02 | .05 | .00 | 1 |  |  |  |  |
|  | **Interventions** |  |  |  |  |  |  |  |  |  |  |  |  |  |  |  |  |  |  |  |  |  |  |  |
| **20** | Adequate counselling | -.02 | .00 | .01 | -.02 | -.05 | .01 | -.04 | .05 | .00 | -.07 | -.**10^*^** | .01 | -.08 | .01 | .**13^*^** | -.05 | .12^*^ | .00 | -.05 | 1 |  |  |  |
| **21** | Encouraged to disclose HIV | .03 | -.12 | .02 | -.**13^**^** | -.09 | **-.10^*^** | **-.16^**^** | .07 | .03 | .00 | .04 | -.10 | -.01 | .07 | .07 | .07 | .**11**^*^ | .03 | **.12^*^** | .07 | 1 |  |  |
| **22** | Adherence supporter | .08 | -.01 | -.06 | -.07 | **-.16^**^** | **-.13^**^** | **-.27^**^** | -.07 | .08 | .08 | .09 | -.01 | -.05 | -.01 | .00 | .02 | -.07 | **.10^*^** | .06 | .09 | .**11^*^** | 1 |  |
| **23** | SM | **-.13^**^** | .07 | **.19^**^** | **.17^**^** | **.15^**^** | **-.11**** | .00 | -.07 | **.19^**^** | .00 | .00 | -.03 | **.13^**^** | **.26^**^** | **.12^*^** | .09 | **.11^*^** | **.15^**^** | **.11^*^** | .03 | **.12^*^** | **.10^*^** | 1 |

*** P-value less than 0.01; * p-value less than 0.05*
