# Supplementary material for: Factors influencing self-management of adults living with HIV on antiretroviral therapy in Northwest Ethiopia: a cross-sectional study
Source: BMC Infect Dis. 2020 Nov 23;20:879. doi: 10.1186/s12879-020-05618-y (PMC7686766; doi:10.1186/s12879-020-05618-y)
Supplement: Supplementary file 2 — Additional file 2 : S2. Survey tool [file 12879_2020_5618_MOESM2_ESM.docx]

**Annex: Data collection tool**

**Date of interview: _______________________________________________ Code: _____________**

**Instruction: *circle the response for the close-ended questions and write responses on the space for the open-ended questions. The tool has six parts.***

| **Part I: Questions on the contextual factors in self-management of adults living with HIV** | | | | | | | | | |
| --- | --- | --- | --- | --- | --- | --- | --- | --- | --- |
| **A** | **Sociodemographic characteristics/ Individual factors** | | |  | | | | | |
| **S.no.** | **Questions** | **Responses** | | **Code** | | | | | |
| 1 | Gender | 1. Male 2. Female | |  | | | | | |
| 2 | Age | ………………. Year | |  | | | | | |
| 3 | Your educational level | 1. No formal education attended 2. Primary education 3. Secondary education (9-10^th^ grade) 4. Preparatory school (11-12^th^ grade) 5. College education 6. University education | |  | | | | | |
| 4 | Your job status | 1. Governmental employed 2. NGO employed 3. Private employed 4. Daily labourer 5. I do not have job | |  | | | | | |
| 5 | What is your income? | ……………. Ethiopian Birr | |  | | | | | |
| B | **Sociodemographic characteristics/ Physical and social environment** | | |  | | | | | |
| **S.no.** | **Questions** | **Responses** | | **Code** | | | | | |
| 6 | What is your religion? | 1. Orthodox Christian 2. Protestant Christian 3. Muslim 4. Other…………… | |  | | | | | |
| 7 | With whom you are living? | 1. Lives alone 2. With family 3. With parents 4. With friends | |  | | | | | |
| 8 | Your marital status | 1. Never married 2. Married 3. Live separated 4. Divorce 5. Widowed | |  | | | | | |
| 9 | Where is your residency? | 1. Rural 2. Urban | |  | | | | | |
| 10 | Distance from this hospital? | ………………. Kilometres | |  | | | | | |
| C | **Condition specific factors** |  | |  | | | | | |
| **S.no.** | **Questions** | **Responses** | | **Code/ skip** | | | | | |
| 11 | When did you diagnosed as HIV positive? | …………………………year | |  | | | | | |
| 12 | For how long you were on antiretroviral therapy? | …………………………………months | |  | | | | | |
| 13 | Your stage of HIV? | 1. Stage I 2. Stage II 3. Stage III 4. Stage IV 5. I do not know | |  | | | | | |
| 14 | Is your treatment changed? | 1. Yes 2. No | | If no, skip to Q16 | | | | | |
| 15 | If yes to Q 14, why your drug is changed? | 1. the drug's side effect 2. I do not know 3. Not taken properly 4. Other (please specify) ………… | |  | | | | | |
| 16 | Do you have any comorbidities? | 1. Yes 2. No | |  | | | | | |
| 17 | Do you take treatment other than antiretroviral therapy? | 1. Yes 2. No | |  | | | | | |
| 18 | How many drugs you are taking for your HIV treatment? | 1. One type 2. Two types 3. Three types | |  | | | | | |
| 19 | Do you have drug side effects? | 1. Yes 2. No | |  | | | | | |
| **Part II: Questionnaire on the process of self-management** | | | | | | | | | |
| 1. **Knowledge about Antiretroviral Therapy** | | | | | | | | | |
| **S.no** | **Variables** | | | **Responses** | | | | | |
| 20 | Are you familiar with your HIV medications? | | | 1. Yes 2. No | | | | | |
| 21 | If you miss your medication dose, do you know what to do? | | | 1. Yes 2. No | | | | | |
| 22 | Are you familiar with the side effects of your medications? | | | 1. Yes 2. No | | | | | |
| 23 | HIV medications can eradicate the virus from your body. | | | 1. Yes 2. No | | | | | |
| 24 | If you feel better, it is ok for you to miss some doses. | | | 1. Yes 2. No | | | | | |
| 25 | Do you know that HIV medications can interact with alcohol? | | | 1. Yes 2. No | | | | | |
| 26 | Do you know skipping HIV medications would really hurt others? | | | 1. Yes 2. No | | | | | |
| 27 | Do you know how HIV medication works in your body to fight HIV? | | | 1. Yes 2. No | | | | | |
| 28 | Do you know if you did not take your HIV medication as ordered, they may not be effective in the future? | | | 1. Yes 2. No | | | | | |
| 29 | Do you know that taking HIV medication as prescribed helps you live longer? | | | 1. Yes 2. No | | | | | |
| 1. **Self-efficacy for HIV Self-Management** | | | | | | | | | |
| **S/no** | **Items** | | **Response** | | | | | | |
|  |  |  | Agree= 3, Neutral= 2 and Disagree=1 | | | | | | |
|  |  |  | **Agree** | **Neutral** | | | | **Disagree** | |
| 30 | It is difficult for me to find effective solutions for problems with managing my HIV infection. | |  |  | | | |  | |
| 31 | I find my efforts to change things I don’t like about my HIV infection are ineffective. | |  |  | | | |  | |
| 32 | I handle myself well with respect to my HIV infection. | |  |  | | | |  | |
| 33 | I succeed in the projects I undertake to manage my HIV infection. | |  |  | | | |  | |
| 34 | I am able to manage things related to my HIV infection as well as most other people. | |  |  | | | |  | |
| 35 | Typically, my plans for managing my HIV infection don’t work out well. | |  |  | | | |  | |
| 36 | No matter how hard I try, managing my HIV infection doesn’t turn out the way I would like. | |  |  | | | |  | |
| 37 | I’m generally able to accomplish my goals with respect to my HIV infection. | |  |  | | | |  | |
| **C** | **Self-regulation abilities** | | **Responses** | **Code/ skip** | | | | | |
| 38 | Do you have plan for self-management of emotional distress? | | 1. Yes 2. No |  | | | |  | |
| 39 | Are you familiar on how to manage your HIV illness related symptoms? | | 1. Yes 2. No |  | | | |  | |
| 40 | Have you set a goal in the process of your HIV therapy? | | 1. Yes 2. No |  | | | |  | |
| **D** | **Social facilitations** | |  |  | | | | | |
| 41 | Have you joined networks of people living with HIV? | | 1. Yes 2. No |  | | | | | |
| 42 | Do you think you have adequately linked to social/ peer network? | | 1. Yes 2. No |  | | | | | |
| 43 | Did the health care providers provide you information on ART? | | 1. Yes 2. No |  | | | | | |
| 44 | Have you disclosed your HIV status? | | 1. Yes 2. No |  | | | | | |
| 45 | If “No” to question 44, what is the reason/s for not disclosing? (more than one is possible) | | 1. Fear of stigma and discrimination 2. Considered not important 3. Other (Specify)… | | | | | | |
| 46 | Do you have reminders for your HIV management? | | 1. Yes 2. No |  | | | | | |
| **E** | **SM Interventions** | |  |  | | | | | |
| 47 | Did you supported by adherence support group? | | 1. Yes 2. No |  | | | | | |
| 48 | Do you think the counselling you got was adequate for your next steps in your HIV treatment? | | 1. Yes 2. No |  | | | | | |
| 49 | Have you been encouraged to disclose your HIV status? | | 1. Yes 2. No |  | | | | | |
| **Part III: Tasks of self-management of adults living with HIV** | | | |  | | | | | |
| **Instruction 2:** *(3= All of the time, 2=some of the time, 1=none of the time, 0=Not applicable, and mark* ***“X”*** *where appropriate.* | | | | | | | | | |
| **S/no** | **Questions** | | | **Responses** | | | | | |
|  |  |  |  | **3** | **2** | **1** | **0** | | **code** |
| **A** | **Domain 1: Daily Health Practice** | | |  |  |  |  | |  |
| 50 | Staying physically active (exercising) is an important part of my HIV management strategy | | |  |  |  |  | |  |
| 51 | I have been successful at staying physically active (walking, exercising, stretching, weight lifting, physical work) | | |  |  |  |  | |  |
| 52 | Spirituality/Religion is my motivator to manage HIV | | |  |  |  |  | |  |
| 53 | I have been changing some aspect of my health to better manage HIV (ex: taking medication, exercising, reducing stress) | | |  |  |  |  | |  |
| 54 | I have been successful at achieving my health goals | | |  |  |  |  | |  |
| 55 | I modified my diet to better manage HIV (vegetables, fruits, natural ingredients) | | |  |  |  |  | |  |
| 56 | Even with all of my family responsibilities I had have enough time to take care of my health needs | | |  |  |  |  | |  |
| 57 | I set aside personal time to do things I enjoy | | |  |  |  |  | |  |
| 58 | My job responsibilities help me to take care of my health | | |  |  |  |  | |  |
| 59 | Educating others about HIV helps me stay in control of HIV (working as a counsellor, advocating for safe sex) | | |  |  |  |  | |  |
| 60 | When I was stressed out I did positive things to relieve the stress (exercising OR journaling OR joining a group) | | |  |  |  |  | |  |
| 61 | I was able to control (or manage) HIV symptoms and medication side effects | | |  |  |  |  | |  |
| **B** | **Domain 2: Resource mobilisation for HIV Self-Management** | | |  |  |  |  | |  |
| 62 | When I feel overwhelmed, I find that talking to my counsellor or attending support groups is very helpful. | | |  |  |  |  | |  |
| 63 | Attending support groups is an important part of HIV Management strategy. | | |  |  |  |  | |  |
| 64 | I have been attending support groups because I found that listening to someone’s testimony or personal story motivates me to take better care of myself. | | |  |  |  |  | |  |
| **C** | **Domain 3: Chronic Nature of HIV and Self-Management** | | |  |  |  |  | |  |
| 65 | I have accepted that HIV is a chronic (or life-long) condition that can be managed | | |  |  |  |  | |  |
| 66 | Managing HIV is a number one priority for me | | |  |  |  |  | |  |
| 67 | HIV has been my motivator to take better care of myself | | |  |  |  |  | |  |
| 68 | I call to make appointments with my HIV doctor when I needed to (change in symptoms, problems with meds, new health concern) | | |  |  |  |  | |  |
| 69 | My HIV doctor and I have a good relationship | | |  |  |  |  | |  |

Thank you for your time contribution.

Name of the interviewer: __________________ Signature: _________ Date: _________
